# Implications of enolase in the RANKL-mediated osteoclast activity following spinal cord injury

**DOI:** 10.32604/biocell.2021.017659

**Published:** 2021-09-01

**Authors:** Ramsha SHAMS, Naren L. BANIK, Azizul HAQUE

**Affiliations:** 1 Department of Microbiology and Immunology, Medical University of South Carolina, Charleston, SC 29425, USA; 2 Department of Neurosurgery, Medical University of South Carolina, Charleston, SC 29425, USA; 3 Ralph H. Johnson Veterans Administration Medical Center, Charleston, SC 29401, USA

**Keywords:** Enolase, RANK, RANKL, Osteoporosis, Neuronal death, Spinal cord injury

## Abstract

Spinal Cord Injury (SCI) is a debilitating condition characterized by damage to the spinal cord, resulting in loss of function, mobility, and sensation. Although increasingly prevalent in the US, no FDA-approved therapy exists due to the unfortunate complexity of the condition, and the difficulties of SCI may be furthered by the development of SCI-related complications, such as osteoporosis. SCI demonstrates two crucial stages for consideration: the primary stage and the secondary stage. While the primary stage is suggested to be immediate and irreversible, the secondary stage is proposed as a promising window of opportunity for therapeutic intervention. Enolase, a metabolic enzyme upregulated after SCI, performs non-glycolytic functions, promoting inflammatory events via extracellular degradative actions and increased production of inflammatory cytokines and chemokines. Neuron-specific enolase (NSE) serves as a biomarker of functional damage to neurons following SCI, and the inhibition of NSE has been demonstrated to reduce signs of secondary injury of SCI and to ameliorate dysfunction. This Viewpoint article involves enolase activation in the regulation of RANK-RANKL pathway and summarizes succinctly the mechanisms influencing osteoclast-mediated resorption of bone in SCI. Our laboratory proposes that inhibition of enolase activation may reduce SCI-induced inflammatory response and decrease osteoclast activity, limiting the chances of skeletal tissue loss in SCI.

## Introduction

Spinal cord injury (SCI) is a condition in which trauma occurs to the spinal cord, resulting in compromised sensation, function, and mobility (Guilcher *et al*., 2019). The pathophysiology of SCI has two phases, a primary phase and a secondary phase. Primary injury involves mechanical trauma, such as compression, laceration, and contusion, and vascular injury to the spinal cord (Alizadeh *et al*., 2019; Cox *et al*., 2015; Hachem *et al*., 2017; Shams *et al*., 2021). Cellular damage at the lesion site is considered immediate and irreversible. In contrast, secondary trauma describes a cascade of molecular events occurring days to weeks following injury, consisting of neuroinflammation, cell death, and tissue degeneration (Hachem *et al*., 2017). SCI-associated inflammation influences the development of chronic ischemic hypoxia, upregulation of Calpain proteases, activation of inflammatory signaling proteins, degeneration of the blood brain barrier, and production of reactive oxygen species (Cox *et al*., 2015; Shams *et al*., 2021). Therefore, the magnitude of the secondary phase of SCI drives the extent of chronic morbidity in individuals with SCI (Cox *et al*., 2015; Hachem *et al*., 2017; Shams *et al*., 2021).

Individuals with SCI are likely to experience paralysis-related complications, such as osteoporosis, which can increase their risk of forming fractures and further the reduction of their quality of life (Hachem *et al*., 2017; Haider *et al*., 2018; Shams *et al*., 2021). Several multidisciplinary mechanisms have been proposed in the literature for their role in bone loss following SCI (Invernizzi *et al*., 2020a). Perturbations in the Wnt pathway have been implicated in bone loss (Shams *et al*., 2021; Zhao *et al*., 2018). The Wnt pathway inhibitor, sclerostin, is upregulated following SCI, significantly compromising bone mineral density, bone strength, and bone architecture in rodent models (Zhao *et al*., 2018). An additional factor in bone loss is mechanical unloading, such as with SCI-induced paralysis, as it has been strongly implicated in the upregulation of sclerostin, as well as the downregulation of periostin, a protein promoting osteoblast activity (Invernizzi *et al*., 2020a; Zhao *et al*., 2018). Mechanical unloading may also upregulate osteoblast secretion of RANKL, contributing to increased bone resorption (Invernizzi *et al*., 2020a; Shams *et al*., 2021). In SCI, mechanical unloading may contribute to muscle atrophy, having crucial crosstalk with skeletal bone tissue; both smooth muscle cells and osteoblasts secrete inflammatory cytokines and chemokines, such as interleukin-6 (IL-6), during physical inactivity (Invernizzi *et al*., 2020a; Invernizzi *et al*., 2020b). Chronic inflammatory condition is suggested to be a major culprit in cellular apoptosis, tissue damage, and oxidative damage (Orr and Gensel, 2018; Samantaray *et al*., 2016).

In the US, 17,730 new SCI cases are reported annually, and unfortunately, this devastating condition has no FDA-approved therapy and hence, no cure (Alabama, 2019). However, this secondary phase is suggested to be reversible, posing it as a promising window of opportunity for therapeutic intervention (Polcyn *et al*., 2017; Shams *et al*., 2021). This short review aims to summarize our viewpoints on the mechanisms influencing osteoporosis or skeletal tissue loss in SCI via enolase-mediated inflammation and promotion of the receptor activator of nuclear factor-κB (RANK) pathway, a modulator for osteoclastogenesis and bone resorption. Our laboratory proposes that enolase inhibition may attenuate activation of RANK ligand (RANKL) and reduce inflammatory response and skeletal loss in SCI.

### Mechanisms of healthy bone remodeling

Bone remodeling is a coupled process between bone construction by osteoblasts and bone resorption by osteoclasts. Transcription factors such as NF-kB, c-Fos, and NFATc1 secreted by osteoblasts stimulate the maturation of osteoclast progenitors. Mature osteoclasts secrete cathepsin K, with the function to dissolve the bone matrix, and hydrochloric acid, with the role to dissolve the bone mineral (Armas and Recker, 2012). When osteoblasts are activated by Wnt signaling proteins, extracellular transmitters (prostaglandin E_2_, prostacyclin, insulin-like growth factor 1, insulin-like growth factor 2, transforming growth factor beta), and intracellular signal transmitters (calcium, inositol trisphosphate (IP3), cyclic adenosine monophosphate (cAMP), and cyclic guanosine monophosphate (cGMP), osteoblasts are immobilized to layer across the dissolved surface of the bone for calcification (Qin *et al*., 2010). When these processes are coupled, there is healthy bone remodeling (Shams *et al*., 2021). Bone remodeling is influenced by mechanical loading onto the bone via weight-bearing, and through biochemical signaling, osteocytes can regulate bone remodeling. Upon injury, these processes may become uncoupled, and the osteocyte’s ability to modulate osteoblast and osteoclast activity is perturbed (Bonewald and Johnson, 2008; Shams *et al*., 2021).

### The RANK/RANKL/OPG pathway and SCI-dysregulation of bone remodeling

The RANK/RANKL/OPG pathway is a modulator for bone resorption (Jiang *et al*., 2014; Liu and Zhang, 2015). This pathway consists of the RANK receptor, RANK ligand (RANKL), and osteoprotegerin (OPG) (Jiang *et al*., 2014; Liu and Zhang, 2015). It is suggested that osteocytes demonstrate RANKL upregulation, activating osteoclastogenesis (Wu *et al*., 2017). RANKL is expressed in osteoblast, osteoblast precursors, and stromal cells (Udagawa *et al*., 1999; Yasuda *et al*., 1998). The RANK receptor is particularly expressed on the surface of osteoclast and pre-osteoclast cells. Osteoblasts or osteocyte cells present RANKL to the RANK receptor embedded on the membrane of osteoclastic progenitor cells (Shams *et al*., 2021). Successful binding of RANKL to the RANK receptor promotes osteoclast differentiation and activation through the stimulation of tumor necrosis factor receptor-associated factor (TRAF6) recruitment (Jiang *et al*., 2014; Liu and Zhang, 2015). TRAF6 recruitment initiates the activation of nuclear factor-κB (NF-κB), p38-extracellular signal-regulated kinase (ERK), and c-Jun NH(2)-terminal kinase (JNK), subsequently interacting with nuclear factor of activated T cells C1 (NFATc1) in the cell nucleus and stimulating the transcription of genes crucial for osteoclastogenesis (Chen *et al*., 2020; Liu *et al*., 2020; Qian *et al*., 2020).

In individuals with SCI, bone remodeling is no longer balanced due to perturbations of the RANK/RANKL/OPG pathway, resulting in osteoporosis (Jiang *et al*., 2014; Kovacs *et al*., 2019). Following SCI, RANK ligand (RANKL) is upregulated, binding to the RANK receptors on osteoclasts (Invernizzi *et al*., 2020b). Osteoclast activation promotes TRAF-6 recruitment and activation of transcription factors JNK, p38, ERK, Akt, IκB, and NF-κB (Chen *et al*., 2020; Kim *et al*., 2018; Lee *et al*., 2005). Phosphorylation of these factors activates the NF-κB, MAPK, and AKT cellular signaling pathways, inducing the increased activation and translocation of NFATc1 and subsequent osteoclastogenesis (Chen *et al*., 2020). Perturbed off-pathway influences have been suggested to dysregulate osteoblast proliferation and maturation. With increased osteoclastogenesis and decreased osteoblastogenesis, bone integrity is compromised, and osteoporosis results (Shams *et al*., 2021). In SCI, bone remodeling may become uncoupled due to SCI-related impairments such as paralysis, malnutrition, or side effects of pharmacological treatments (Jiang *et al*., 2006). SCI may stimulate osteoclastogenesis by promoting the secretion of IL-6, whose role in osteoclast cell fate determination has been implicated (Demulder *et al*., 1998; Francois *et al*., 2006; Kurihara *et al*., 1990).

### Enolase

Enolase activation has been implicated in SCI (Haque *et al*., 2016). Enolase is a highly conserved, glycolytic enzyme with ubiquitous expression; it functions to convert 2-phosphoglycerate to phosphoenolpyruvate (Day *et al*., 1993). Several enolase isoforms exist, both as homodimers and heterodimers of the α-, β-, and γ-enolase subunits (Marangos *et al*., 1978; Shimizu *et al*., 1983). While enolase primarily functions in glycolysis, it also exhibits nonglycolytic functions (Avilan *et al*., 2011). When expressed in the cytosol, enolase interacts with the cytoskeleton to mediate material trafficking and influence cell morphology (Keller *et al*., 2007). Enolase expression in the nucleus is implicated with the regulation of genes of cell proliferation and morphological transformation (Lung *et al*., 2010). In conditions leading to cellular injury, enolase appears on the cell surface and induces a cascade of inflammatory immune responses via the activation of the CD14-dependent Toll-Like Receptor 4 (TLR4) pathway and downstream activation of NF-κB, eliciting powerful stimulation of pro-inflammatory cytokines and chemokines (Guillou *et al*., 2016; Haque *et al*., 2016). NF-κB may stimulate IL-1β (interleukin-1β), tumor necrosis factor alpha (TNF-α), IL-6 (interleukin-6), macrophage colony-stimulating factor (m-CSF), and additional inflammatory molecules (Barnes and Karin, 1997) . These may contribute to reactive oxygen species (ROS) production, exasperated cellular functioning, and tissue degeneration (Barnes and Karin, 1997; Haque *et al*., 2017).

### Neuron specific enolase

Neuron specific enolase (NSE) is used as a marker for neurons and neuroendocrine neurons, and studies suggest NSE may perform inflammatory and neurotrophic activities, mediating neuronal differentiation, proliferation, survival, and death (Bae *et al*., 2012; Hafner *et al*., 2012; Haque *et al*., 2018). Elevated levels of enolase may induce cellular death and tissue degeneration through the production of reactive oxygen species (ROS), nitric oxide (NO), and pro-inflammatory molecules (Haque *et al*., 2017; Haque *et al*., 2016; Sedoris *et al*., 2010). NSE is implicated in conditions involving ischemia, hypoxia, and inflammation, such as autoimmune and neuroinflammatory diseases. Although NSE is required for neuronal survival, high levels of NSE could be detrimental (Anand and Stead, 2005; Woodcock and Morganti-Kossmann, 2013). In further, increased secretion of NSE in serum and cerebrospinal fluid (CSF) is implicated with a higher magnitude of neurodegeneration and subsequent disease progression (Haque *et al*., 2018). In studies using SCI models, it has been suggested that rats with SCI have elevated NSE levels than rats with no SCI, and the inhibition of NSE has dual functions in secondary injury of SCI (Haque *et al*., 2017; Li *et al*., 2014).

### Enolase inhibition

The Haque lab proposed inhibition of enolase activation as an effective strategy against the detrimental inflammatory events in SCI (Haque *et al*., 2016; Polcyn *et al*., 2020). ENOblock is a novel, small enolase inhibitor, developed to study predominantly the non-glycolytic functions of enolase. In an SCI rat model, it has been demonstrated that ENOblock can attenuate inflammatory metabolic and signaling factors (Li *et al*., 2014; Polcyn *et al*., 2017). ENOblock administration can result in the inhibition of Iba-1/GFAP expression (gliosis), reducing pro-inflammatory events in SCI rat models. ENOblock may also reduce NSE and subsequent pro-inflammatory cytokine/chemokine levels in serum and spinal cord tissue (Haque *et al*., 2017).

### Potential enolase and RANK/RANKL pathway interactions

**FIGURE 1.**
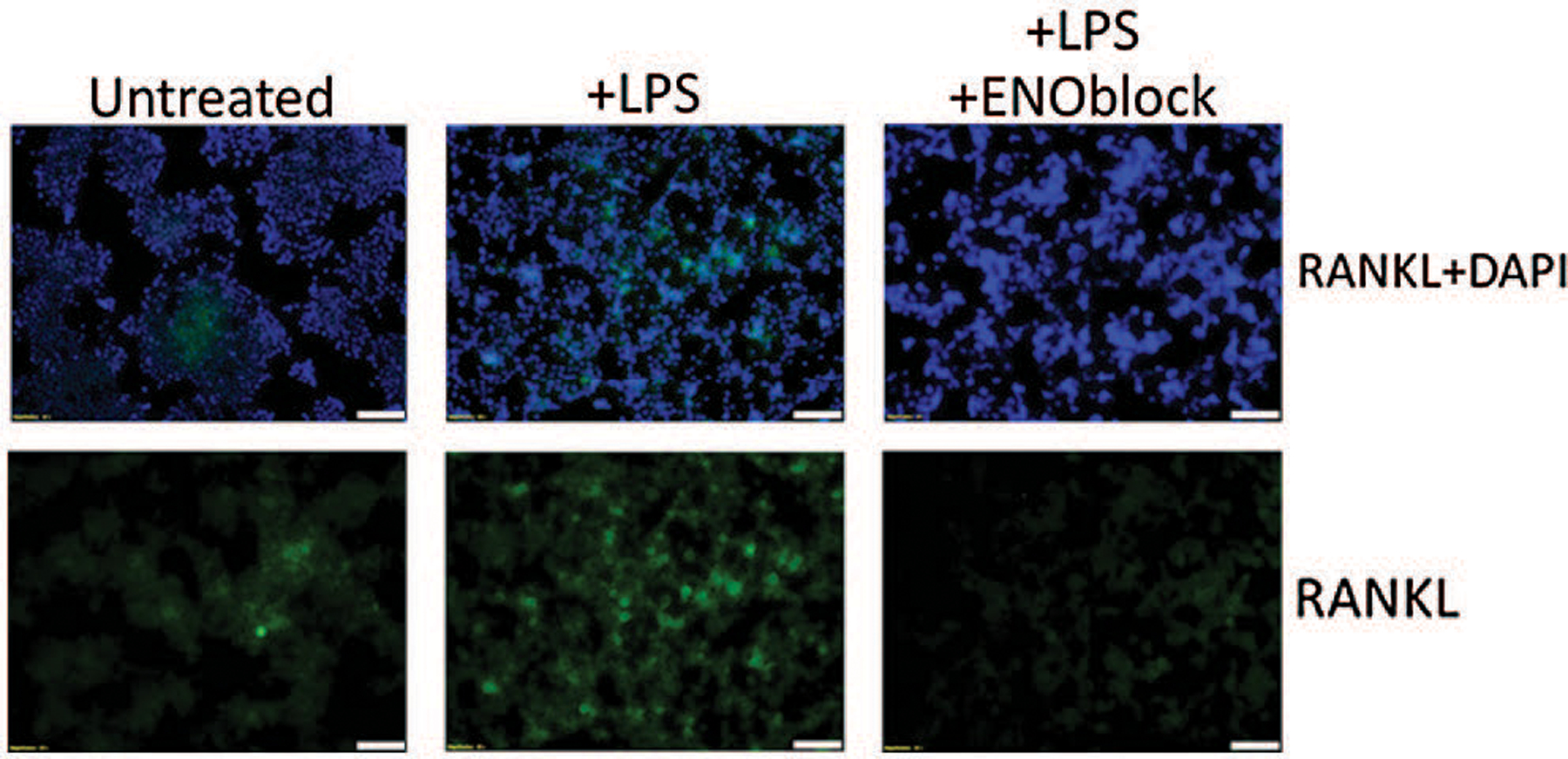
Enolase inhibition decreases RANKL expression in Raw264.7 cells. Raw264.7 macrophage cells were cultured in Dulbecco’s Modification of Eagle’s Medium (DMEM) with 4.5 g/L D-Gluvose, L-Glutamine, and 110 mg/L Sodium Pyruvate (11995–065, Gibco), supplemented with 10% Fetal Bovine Serum (FBS) (Thermo Scientific, Logan) and Penicillin-Streptomycin (P/S) (Mediatech, Inc., Herndon). Cells were treated with vehicle alone, LPS (100 ng/mL), and LPS + ENOblock (2.5 μM) for 72 hours. Immunofluorescence staining was performed to detect RANKL expression (sc-377079, Santa Cruz). Nuclear staining was performed using DAPI. Representative images of three trials are presented in 20x magnification.

**FIGURE 2.**
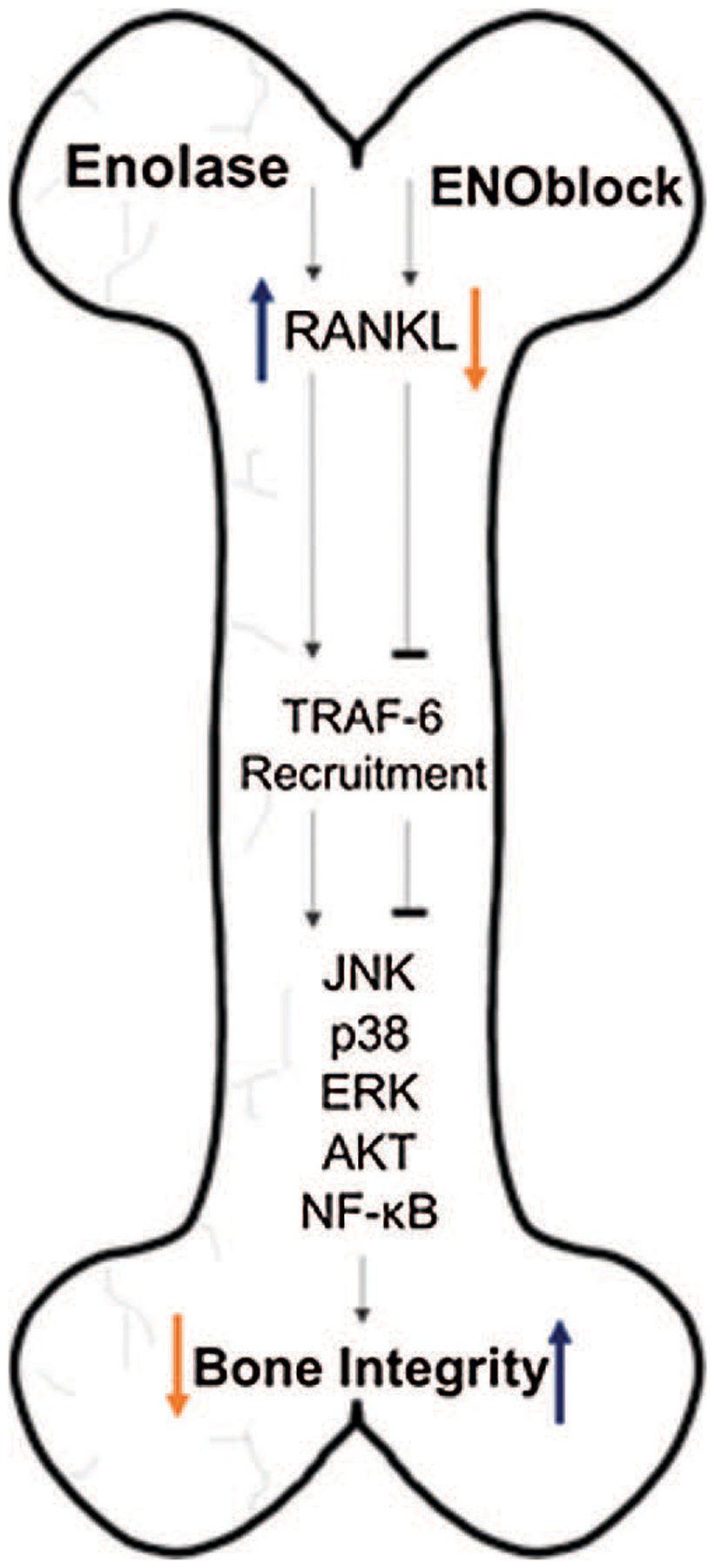
Enolase activation may trigger inflammatory responses leading to upregulation of RANKL after SCI. RANKL upregulation induces TRAF-6 recruitment, which in turn, promotes the activation of the transcription factors JNK, p38, ERK, Akt, and NF-κB. This causes an increase in osteoclast activity, leading to compromised bone integrity. Inversely, inhibition of enolase activation via ENOblock may block the upregulation of RANKL, and TRAF-6. Since the respective transcription factors would not be activated, osteoclast activity may be mitigated, preserving bone integrity.

LPS-stimulated cells may experience pro-inflammatory conditions via production of interferon gamma (IFN-γ) and tumor necrosis factor alpha (TNF-α) secretion (Guillou *et al*., 2016). α-enolase or ENO1 is preferentially expressed by monocyte and macrophage cells, Raw264.7 cells for example. α-enolase-stimulated cells experience similar pro-inflammatory response to LPS-stimulated cells (Guillou *et al*., 2016). It is suggested that, in the inflammatory process, cell-surface expression of α-enolase is dramatically upregulated in monocytes following LPS-stimulation, due to the translocation of ENO1 to the surface of the cell. Studies in our laboratory suggest that, when enolase is inhibited, the inflammatory process is perturbed in LPS-stimulated Raw 264.7 cells as previously suggested (Guillou *et al*., 2016). In the inflammatory state, LPS-stimulated Raw264.7 cells, a cellular model of osteoclast activity, demonstrate up-regulated RANKL expression. With enolase inhibition via ENOblock, we demonstrate that RANKL expression is decreased compared to untreated and LPS-stimulated Raw264.7 cells (Fig. 1). We suggest that, with enolase inhibition, osteoclast activation via RANKL binding can be perturbed following SCI, thereby preventing the recruitment of TRAF-6 and activation of JNK, p38, ERK, Akt and NF-κB (Fig. 2). Therefore, inhibition of enolase activation could mitigate SCI-induced osteoporosis or skeletal loss.

### Study limitations

The data is representative of preclinical studies using *in vitro* model. However, several multifactorial mechanisms influencing bone loss following SCI are described in the literature and may significantly skew the plausibility of these results. Additionally, with the novel nature of the study, the lack of systematic database search may hinder the reproducibility of the results and the strength of the conclusions.

## Conclusion

This viewpoint short review attempts to summarize mechanisms mediating skeletal loss in SCI via osteoclast-mediated resorption of bone and the role of enolase activation on the RANK/RANKL pathway. This is the first viewpoint summarizing the current literature on this topic, aiming at paving the way to future works on this novel promising therapeutic target.
